# Gut Microbiota, Antibiotic Therapy and Antimicrobial Resistance: A Narrative Review

**DOI:** 10.3390/microorganisms8020269

**Published:** 2020-02-17

**Authors:** Benoit Pilmis, Alban Le Monnier, Jean-Ralph Zahar

**Affiliations:** 1 Équipe mobile de Microbiologie clinique, Groupe Hospitalier Paris Saint Joseph, 75014 Paris, France; 2 Service de maladies infectieuses et tropicales, Hôpital Necker Enfants-Malades, 75015 Paris, France; 3 Université Paris-Saclay, INRAE, AgroParisTech, institut MIcalis, 92290 Chatenay-Malabry, France; alemonnier@hpsj.fr; 4 Laboratoire de Microbiologie Clinique et Plateforme de dosage des anti-infectieux, Groupe Hospitalier Paris Saint Joseph, 75014 Paris, France; 5 Service de Microbiologie Clinique et Unité de Contrôle et de Prévention du risque Infectieux, Groupe Hospitalier Paris Seine Saint-Denis, AP-HP, 125 rue de Stalingrad, 93000 Bobigny, France; jrzahar@gmail.com; 6 IAME, UMR 1137, Université Paris 13, 75890 Sorbonne Paris Cité, France

**Keywords:** gut microbiota, antimicrobial resistance, multidrug resistant pathogens

## Abstract

Antimicrobial resistance is a major concern. Epidemiological studies have demonstrated direct relationships between antibiotic consumption and emergence/dissemination of resistant strains. Within the last decade, authors confounded spectrum activity and ecological effects and did not take into account several other factors playing important roles, such as impact on anaerobic flora, biliary elimination and sub-inhibitory concentration. The ecological impact of antibiotics on the gut microbiota by direct or indirect mechanisms reflects the breaking of the resistance barrier to colonization. To limit the impact of antibiotic therapy on gut microbiota, consideration of the spectrum of activity and route of elimination must be integrated into the decision. Various strategies to prevent (antimicrobial stewardship, action on residual antibiotics at colonic level) or cure dysbiosis (prebiotic, probiotic and fecal microbiota transplantation) have been introduced or are currently being developed.

## 1. Introduction

The emergence of antibiotic resistance constitutes a major public health threat. Epidemiological studies have clearly demonstrated direct relationships between antibiotic consumption and the emergence/dissemination of resistant strains in hospitals and intensive care units (ICUs) [1,2,3]. However, antibiotic consumption is not the only factor associated with the spread of resistance. For several years we have been promoting antibiotic de-escalation by basing our thinking on the ecological effect of antibiotic classes. However, while the antibiotic class by its spectrum of activity probably has a role in the emergence of resistance, it alone does not seem to explain the spread of resistance. Indeed, how to explain the discrepancies between studies on the antibiotic effects of the same antibiotic class [4,5], how to underline the *paradoxical* effects of some antibiotic classes described by several authors [6,7]. Finally, how can we explain the lack of impact of our stringent policies of de-escalation at the individual level? Within the last decade, authors confounded spectrum activity and ecological effects [8] and did not take into account several other factors that play important roles, such as impact on anaerobic flora [9], biliary elimination [10,11] and sub-inhibitory concentration [12]. These data have recently led us to better understand the role of gut microbiota on the emergence of resistance.

Gut microbiota is essential for the proper development of the intestinal tract and maturation of the immune and nervous system. In fact, an intact, fully developed gastrointestinal (GI) tract microbiota also protects the host against invasion by pathogenic microorganisms [13,14,15] through a highly complex set of events known as *colonization resistance* [16,17]. Consequently, alteration of the microbiota composition (called dysbiosis) induced by many factors, including antibiotic therapy, can lead to pathology, including asthma and infectious disease [18]. Recent promotion of antimicrobial stewardship and optimization with antibiotic prescriptions, particularly on Pharmacokinetic/Pharmacodynamic (Pk/Pd) parameters, could limit the impact on the gut microbiota. The implementation of these measures has been shown to significantly reduce hospital costs and the use of antibiotics [19]. However, at an individual level, few studies have shown a link between a decrease in broad-spectrum antibiotic consumption and a decrease in antimicrobial resistance [20,21,22]. A study conducted in an intensive care unit found no decrease in the rate of global multidrug resistant (MDR) strain carriage acquisition after de-escalation of pivotal beta-lactam in ventilator-associated pneumoniae [23]. Reducing antibiotic consumption is an absolute necessity. However, promoting de-escalation for infected patients may expose them to the risk of therapeutic failure [8]. So, antimicrobial stewardship needs to be rethought on a broader mission than saving broad-spectrum antibiotics. Actions must integrate Pk/Pd data, therapeutic drug monitoring, impact of antibiotics on anaerobic flora and reduction of treatment duration.

In this narrative review, we propose to provide some consideration, allowing prescribers to put the message into perspective with regard to the risk. We proposed to present (i) what a “normal gut microbiota is”, (ii) discuss *colonization resistance*, (iii) present data concerning the interaction between the gut microbiota and antibiotics, in particular, the Pk/Pd parameters of the latter, (iv) infectious risk related to dysbiosis, (v) manipulations of the intestinal microbiota as a therapeutic approach, (vi) to conclude concerning the role of different antibiotic classes as a promoter of resistance.

## 2. Strategy for Data Search

Data were obtained from articles published in English belonging to journals indexed in PubMed. We also searched the reference lists of retrieved papers for further relevant articles. There was no restriction regarding the date of publication, and we included studies up to November 2019.

## 3. Gut Microbiota Composition

The human gut microbiota refers to the microbes (bacteria, fungi, archaea, viruses, and protozoans) that reside inside the gut [24] and contribute in several functions beneficial to the hosts, including fermentation of food items [25], synthesis of vitamins and amino-acids [26], prevention of colonization by enteropathogenic bacteria [27], maturation and regulation of the immune system [28], modulation of gastrointestinal hormone release and regulation of brain-behavior.

The normal human gut microbiota includes two major (Bacteroidetes and Firmicutes) and two minor (Actinobacteria and Proteobacteria) phyla. Even though this general profile remains constant, gut microbiota exhibits both temporal and spatial differences in distribution at the genus level and beyond according to pH and aerobic conditions. As one travels from the distal esophagus distally to the rectum, there will be a marked difference in diversity and number of bacteria ranging from 10^1^ bacteria per gram of contents in the esophagus and stomach to 10^12^ bacteria per gram of contents in the colon and distal gut. The predominant phyla that inhabit the large intestine include *Firmicutes* and *Bacteroidetes*. Conventionally, the *Firmicutes/Bacteroidetes* ratio has been implicated in predisposition to disease state as obesity [29] even though the increase in the relative abundance of Proteobacteria (such as *Escherichia coli* and *Klebsiella pneumoniae*) could be a sign of dysbiosis and an increased risk of infection due to the rupture of the colonization resistance barrier [30]. Besides these longitudinal differences, there also exists an axial difference from the lumen to the mucosal surface of the intestine. While *Bacteroides*, *Bifidobacterium*, *Streptococcus*, *Enterobacteriaceae*, *Enterococcus*, *Clostridium*, *Lactobacillus* and *Ruminococcus* are the predominant luminal microbial genera (can be identified in stool), only *Clostridium*, *Lactobacillus*, *Enterococcus*, and *Akkermansia* are the predominant mucosa and mucus associated genera (detected in the mucus layer and epithelial crypts of the small intestine) [31].

The development of culture-independent, high-throughput molecular techniques have enabled the identification of previously unknown bacterial species, thereby providing novel insights into the compositional diversity and functional capacity of fecal microbiota. To this end, two concepts of diversity have been proposed: overall fecal microbiota structure, that is, richness, abundance, evenness individual (α-diversity) and compositional dissimilarity (β-diversity). These two notions are important to characterize the effect of a drug or those of probiotics on the gut microbiota. Indeed, interpretation of an effect on the composition of gut microbiota in healthy individuals may be particularly complicated due to the lack of an internationally accepted consensus definition of healthy or abnormal fecal microbial community [32]. 

The collective genes that an individual’s gut microbiota encompasses are known as the microbiome. It overwhelmingly surpasses the coding capacity of the human genome with more than three million genes [33]. Although there is large inter-individual variability in the bacterial species comprising the host’s microbiota, many microbial genes share functions, resulting in high functional redundancy between microbiomes, thus composing the “core microbiome”. These genes act, for example, on the digestion of complex sugars and glycans, synthesis of amino acids, detoxification of xenobiotics [34].

## 4. Colonization Resistance

### 4.1. Concept of Colonization Resistance

Gut microbiota plays a crucial role in excluding invading exogenous bacteria and inhibiting the overgrowth of indigenous under dominant bacteria within the intestinal tract. The role of the microbiota in host defense against enteric pathogens was first described in 1954 by Bohnoff et al. [35]. In this work, streptomycin orally administered disrupted the gut microbiota and increased the sensitivity of mice to *Salmonella enterica* subsp. enterica related infections. Other studies involving animals or humans with different antibiotics or pathogens have found similar results [36,37,38]. For example, patients on broad-spectrum antibiotics markedly decrease the levels of protective gut microbiota and allow the proliferation of *Clostridioides difficile* that can be found in low levels in some individuals [39]. This protective role of the gut microbiota against the implantation of enteric pathogens and subsequent infections has been called *colonization resistance* [40]. 

### 4.2. Mechanisms of colonization resistance

It has been known for over 50 years that commensal anaerobes confer protection against exogenous pathogens [17]. In their study, Léonard et al. showed that ceftriaxone had different effects on the fecal flora of volunteers. They demonstrated that the failure of ceftriaxone to modify the fecal flora from some volunteers resulted from the degradation of the antibiotic by β-lactamase producing anaerobic bacteria [41].

#### 4.2.1. Direct Mechanisms

Direct mechanisms of colonization resistance are characterized by the gut microbiota ability to restrict colonization by enteric pathogens, and/or the overgrowth of these pathogens after implantation, and those independent of the host.

##### Nutrient Competition

Mucins and dietary complex carbohydrates are essential intestinal nutritive resources to which commensal species have adapted through specific metabolic pathways [42]. Enteropathogens frequently use nutritious sources offered by commensal species; pathogenic bacteria that are unable to metabolize these sources are frequently eliminated. Indeed, there is a competition between commensal bacteria and pathogenic bacteria for nutrients. For example, *E. coli* and *Citrobacter rodentium* may be in competition for the metabolism of monosaccharides. In the presence of commensal bacteria that can use a wide range of sugar, metabolic pathways can be redirected to allow competition between commensal and pathogenic bacteria [43]. However, some pathogenic bacteria may use digestive nutrients that commensals cannot metabolize. Thus, ethanolamine is a carbon and nitrogen source for *S. Typhimurium*, Entero-Haemorrhagic *E. coli* (EHEC), *Klebsiella*, *Pseudomonas*, *C. difficile* and *Listeria monocytogenes* but cannot be used by most commensal species [44]. EHEC species, in particular, have developed metabolic pathways for distinct sugar resources, some of which are inaccessible to commensal *E. coli* [45]. Interestingly, in the presence of two different strains of commensal *E. coli,* EHEC can be removed from its metabolic niche and could fail to colonize the gut [46]. Furthermore, *Clostridioides difficile* growth is dependent on sialic acid. It has been shown that in conventional mice with a complex microbiota, the sialic acid concentration is low. Ng et al. showed in their work that previous antibiotics administration increases sialic acid concentrations and promotes the multiplication of *Clostridioides difficile* [47].

##### Bacteriocin

The commensal microbiota produces cells wall-active bactericidal polypeptides generically called bacteriocins. These are two types of peptides synthesized by ribosomes: peptides with post-transduction modification (type I) and unmodified peptides (type II) [48]. Many bacteriocins have high specific activity against clinical targets (including MDR strains), have a mechanism of action that is distinct from current chemotherapeutic drugs. Mechanisms of action of bacteriocins can be broadly divided into those that are primarily active on the cell envelope and those that are active primarily within the cell, affecting gene expression and protein production. Although several broad-spectrum bacteriocins exist that can be used to target infections of unknown aetiology, the majority of bacteriocins have a narrow spectrum targeting specific pathogens without negatively affecting the commensal microbiota [49,50]. Thus the bacteriocins produced by Gram-negative bacteria are called microcins and have a narrow spectrum of activity limited to other Gram-negative bacteria [51]. Microcins often target cells expressing the same nutrient receptor (as siderophore) causing its internalization and thus exerting its inhibitory effect [52,53]

The potency of bacteriocins against clinically relevant pathogens has been evaluated in in vitro and in vivo studies. For example, the sactibiotic thuricin CD (bacteriocin type I) has been found to be particularly potent against *C. difficile* [49] and another sactibiotic, the subtilosin A, displays a narrow spectrum of activity against *Enterococcus faecalis*, *Streptococcus pyogenes* and *Listeria monocytogenes* [54]. Otherwise, *Pediococcus acidilactici* MM33 produces pediocin PA-1 (bactericin type II) with activity against Vancomycin-resistant Enterococci (VRE), reducing digestive colonization by this bacteria [55]. 

Clinical applications of bacteriocins seem to be real but will depend on our understanding of their mechanisms of action; however, the risk of emergence of resistance to these peptides have been already described and seems to be due to reduced accessibility to the receptor [56] and/or changes in cell envelope composition [57,58].

##### Type VI secretion System

Secretion systems allow bacteria to transport macromolecules such as protein out of effector cells or into either target host cells during pathogenesis or target bacterial cells during competition in various ecological settings [59]. The effector protein is most often an antimicrobial toxin and most often uses a dependent contact bacterial antagonism mechanism [60]. Several enteric pathogens have T6SSs as *Salmonella enterica* [61], *Citrobacter rodentium* [62], *Aeromonas hydrophila* [63] and enteroaggregative *E. coli* (EAEC) [64]. Furthermore, more than 50% of all *Bacteroides sp.* have *T6SSs* [65]. Gram-positive bacteria are not known to be targeted by T6SSs, but Bacteroidetes and Proteobacteria can potentially be involved in T6SSs. This system would, therefore, be used to defend endogenous bacteria against the overgrowth of certain exogenous pathogenic bacteria species.

#### 4.2.2. Indirect Mechanisms

Interaction of commensal flora with the host results in indirect colonization resistance through antimicrobial peptides production (RegIIIγ and angiotensin-4), epithelial barrier maintenance and bile acid metabolism.

##### Antimicrobial Peptide Production

Antimicrobial peptides (AMPs) are produced by every living organism [66] and are considered as an important line of defense against invading pathogens [67,68]. AMPs exhibit a great number of fundamentally different activities; indeed, most antimicrobial peptides target the bacterial wall and peptidoglycan [67]. The bacterial specificity of antimicrobial peptides is due to the difference in composition between bacterial and eukaryotic membranes [67]. Bacterial membranes are composed of cardiolipin and phosphatidylglycerol, resulting in a negative charge, while most antimicrobial peptides are positively charged, allowing interaction with the bacterial wall and its lysis [69,70]. For example, antimicrobial peptides, such as RegIIIγ (type C lectin) and ANG-4 (ribonuclease), are proteins produced by the host (Paneth cells and epithelial cells) via taurine or LPS [71,72]. MyD88-mediated signal-induced the bacterial lectin RegIIIγ and protect mice against intestinal *Listeria monocytogenes* and VRE colonization/infection [73,74] while ANG-4 expression is induced by *Bacteroides thetaiotaomicron,* a predominant member of the gut microbiota and has bactericidal activity against Gram-negative and Gram-positive bacteria [75].

The AMPs productions are, therefore, regulated by the microbiota. Thus, the microbiota can stimulate RegIIIγ production by stimulating Toll Like Receptors (TLRs), especially TLR-4, by the lipopolysaccharide (LPS) [71]. Furthermore, flagellin stimulates TLR-5 in dendritic cells (TLR5+ CD103+) and TLR7 in dendritic cells (TLR7+CD11c+) [76,77]. Stimulation of these dendritic cells results in the release of IL-23 activating the innate lymphoid cells that secrete IL-22, increasing RegIIIγ production [76].

Taurine (microbial metabolite) via an inflammasome complex in the intestinal epithelium leads to negative regulation of the production of pro-inflammatory cytokines including IL-18. IL-18 promotes the production of antimicrobial peptides, such as ANG-4 [75].

##### Epithelial Barrier Maintenance

The physical gut barrier consists of the inner and outer mucus layer, the epithelial barrier, and its related immune barrier. Indeed, intestinal epithelium from healthy patients is covered with mucus that is poor in bacteria. The inner mucus layer is impenetrable and strongly attached to the epithelium that does not allow bacteria to reach the epithelial cells, thus limits direct contact between the host and commensal bacteria of the gut microbiota, preventing a possible inflammatory response linked to the latter [78,79]. Commensal gut microbiota resides and metabolizes nutriments in the outer mucus layer. Bacterial exposure is associated with the production of a functional mucus barrier as demonstrated by a germ-free animal in which the inner mucus layer is thin but can be restored by exposure to bacterial components [80]. Therefore, a decrease in the thickness of the mucus layer exposes to increased susceptibility to pathogen colonization. The latter is related to the composition of the intestinal microbiota. Thus, the western-style diet, which is deficient in microbiota-accessible carbohydrates [81], antibiotic therapy or other drugs impacting the microbiota, results in a modification of the thickness of the mucus layer and increased susceptibility to colonization/infection. In the case of alteration of the mucus layer, the NF-κB pathway is involved in the cellular damage repair. The intestinal microbiota by stimulating the receptors of innate immunity leads to the activation of this pathway and promotes tissue repair [15]. The NF-κB pathway operates in (i) leading to negative regulation of pro-inflammatory cytokine production, (ii) promoting the production of anti-apoptotic factors, (iii) stimulating cell proliferation, (iv) stabilizing tight junctions [82].

For example, the administration of TLR-5 agonists in mice pretreated with antibiotics and challenged by *C. difficile* leads to a decrease in colonization and toxin production by *C. difficile*. Furthermore, the analysis of the mouse caecum shows better integrity of the epithelial barrier [83].

##### Bile Acid Metabolism

Intestinal microbiota also interacts with host molecules other than the host’s immune system, such as biliary acids. Primary bile acids are reabsorbed in the terminal ileum, but a small remaining fraction reaches the large intestine where a subset of bacteria in the colon can convert them into secondary bile acids. Importantly, different bile acids have different effects on promoting germination and vegetative growth. While the primary bile acid taurocholic acid induces the germination of *C. difficile* spores, secondary bile acids have found to inhibit the growth of vegetative, toxin-producing *C. difficile* [84]. For example, *Clostridium scindens*, a commensal inhabitant of human gut microbiota is able to convert the primary bile acids, cholic acid (CA) and chenodeoxycholic acid (CDCA) to secondary bile acids, deoxycholic acid (DCA) and lithocholic acid (LCA), respectively. Thus, *C. scindens* enhances resistance to *C. difficile* infections in both animal models and in human patients by the way of secondary bile acid-dependent fashion [85].

## 5. Biases in the Interpretation of Studies on the Ecological Impact of Antibiotics

Interpretation of studies concerning the emergence of resistance is complicated because several factors contribute to the emergence of resistance. This explains the discrepancies between studies on antibiotic effects. For example, in their study Grohs et al. found ecological benefits of substitution from ceftriaxone to cefotaxime [11], while a recent study in healthy volunteers showed no difference between the two third-generation cephalosporins [86]. This difference between the results can be explained by methodological considerations. Grohs et al.’s study is a before–after study and probably illustrates one of the limiting principles of before and after studies, the control of confounding factors. Unmeasured variables such as improved hand hygiene, implantation of an antimicrobial stewardship team and attention to contact precautions may have contributed to the decline in the AmpC-producing *Enterobacteriaceae* incidence observed in that study.

Moreover, even though most consider carbapenems to be molecules with a high ecological impact, a study published by Grall et al. showed that colonization rate by imipenem-susceptible ESBL producing *Enterobacteriaceae* remain stable after treatment by imipenem [87]. The authors seem astonished by this finding, even though work in the 1980s revealed a digestive concentration of imipenem in the healthy volunteer with ≤ 2% of the plasma concentration [7] and a relatively modest effect on the bowel flora without inducing resistance in the resident flora [5]

This lack of correlation between antibiotic spectrum, route of administration and ecological impact was also suggested in a study by Connelly et al. [88]. In this study, administration of oral amoxicillin and intravenous ertapenem caused significant alterations in the composition of the microbiome resulting in loss of some species and outgrowth of others in addition to changes in the resistome, the collection of antibiotic resistance genes in the gut microbiome. These studies suggest that the impact of antibiotic therapy on gut microbiota could not be predicted solely based on the spectrum of activity or route of administration of an antibiotic. For conclusion, careful reading of the literature concerning antimicrobial use and resistance should take heed of the potential pitfalls in interpretation: study design (case-control, before–after, randomized control trial, meta-analysis), definition of exposure, analysis of a class of antibiotic or of an antibiotic within a class, route of administration of antibiotics [89], the existence or not of a comparator, outcome measures and metrics used. Awareness of all the elements is essential for the interpretation of studies examining the effect of antibiotic use on resistance.

## 6. From Colonization to Infection

Numerous studies suggest a preliminary colonization step as a mandatory prerequisite for the development of infection related to MDR *Enterobacteriaceae* [78]. Furthermore, only a few studies focused on the risk factors associated with MDR *Enterobacteriaceae* in previously colonized patients [90,91]. In a retrospective case-controlled study, conducted out of ICU and including pediatric and adult patients, authors identified two factors associated with ESBL producing an *Escherichia coli* related infection in previously colonized patients. These factors were the use of a β-lactam/β-lactamase inhibitor prior to infection and urinary catheterization [92]. Therefore, it seems to have an impact of antibiotic therapy on the gut microbiota and a link between the gut quantity of MDR *Enterobacteriaceae* and urinary tract infections occurrence [93].

### 6.1. Digestive Colonization by Multidrug-resistant Bacteria and Gut Microbiota

Previous colonization seems to be the main condition for the occurrence of MDR Enterobacteriaceae related infections. However, studies evaluating the duration of intestinal colonization by MDR bacteria found that duration was very variable from a patient to another.

Some factors seem to be associated with the duration of intestinal colonization such as bacterial species [94,95,96], microbiota composition [41,97,98,99], previous non-antibiotic drugs [100] and previous antibiotic therapy [89,101,102,103].

### 6.2. Drugs Interaction with Human Gut Microbiota

Numerous studies have confirmed that antibiotics have an impact on the composition and functionality of the human microbiota [104]. The impact of antibiotics on the microbiota can lead to (i) selection of resistant bacteria [105,106], (ii) domination of microbial composition by pathogenic bacteria [107], (iii) loss of bacterial diversity [108], (iv) decrease or even loss of certain bacterial species [109], (v) increase in susceptibility to infections and (vi) risk of new infection and/or recurrence.

The ecological consequences of a given antibiotic class depend on drug concentration reaching the gut microbiota and the susceptibility of bacterial species. Furthermore, temporal disorders of the microbiota following antibiotic use appear to persist over time and can reach up to two years in patients treated with macrolides/lincosamides [110,111]. Similar patterns of long-term changes in composition and diversity of gut microbiota have also been observed with amoxicillin and cefpodoxime proxetil orally administered [112]. The expanding bacterial populations during early recovery of the gut microbiota carried across studies and depended on the initial gut microbiota composition. Generally, among patients undergoing antibiotic therapy, the expansion of members from the Proteobacteria phylum was common and could be a signature marker of dysbiosis [112,113,114,115,116].

Integration of Pk/Pd data seems necessary to interpret the impact of an antibiotic on the gut microbiota. Indeed, antibiotics can only alter gut microbiota composition by direct exposure. So, absorption sites of orally administered antibiotics must be taken into account. For example, orally administered metronidazole is almost entirely absorbed in the small intestine resulting in low residual concentrations in the distal digestive tract, explaining some therapeutic failures despite the lack of resistance to metronidazole described in *Clostridioides difficile* [117,118]. This also suggests that along the gastrointestinal tract, oral metronidazole may have less of an impact on the gut microbiota than oral vancomycin (non-absorbed drug). It is well accepted that antibiotics with biliary elimination have the greatest ecological impact. The biliary elimination route is more common with lipophilic agents (fluoroquinolones, macrolides, metronidazole, streptogramins, tetracyclines) [119]. Finally, the impact of an antibiotic on the gut microbiota is also associated with its spectrum of activity, in particular, its impact on anaerobic bacteria. For instance, metronidazole and clindamycin both target numerous anaerobic bacteria, but clindamycin also targets Gram-positive bacteria explaining the more pronounced impact in reducing microbial diversity in the long term [120,121]. Antibiotic’s spectrum of activity can also play an important role in which pathogenic bacteria can consequently colonize and expand within the intestine. Metronidazole treatment results in increasing the risk of intestinal enterococcal colonization and expansion, whereas intravenous vancomycin and beta-lactam administration did not increase the risk [122].

Nowadays, while antibiotic resistance has become a major public health problem, the choice of antibiotic therapies should take into account their “ecological impact”. It, therefore, seems important to weigh the current policy of de-escalation of antibiotics against the ecological impact of the chosen antibiotic alternatives and the risk of therapeutic failure. For example, we could question the relevance of choosing as alternatives to carbapenems in the treatment of ESBL-producing *Enterobacteriaceae* related infections, antibiotic classes with anti-anaerobic activity, such as cephamycins or piperacillin-tazobactam.

To this end, consideration of the spectrum of activity and route of elimination must be integrated into the decision. In the future, for each new antibiotic, ecological impact studies of new antibiotics should be performed before their commercialization.

## 7. How to Minimize Antibiotic Therapy Impact on Gut Microbiota

### 7.1. Strategy to Prevent the Occurrence of Dysbiosis

**Figure 1:**
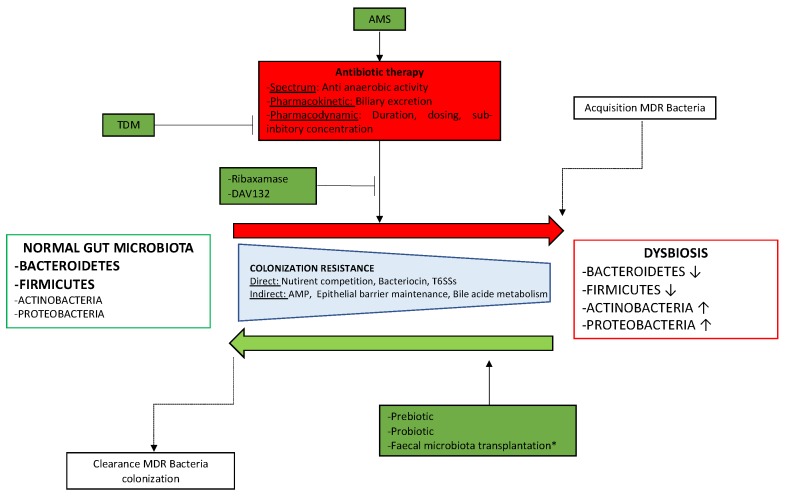
Impact of antibitioc therapy on gut microbiota and management of dysbiosis.

About 25% of hospitalized patients received antibiotics. Approximatively a third to half of the antibiotic therapy prescriptions are issued with too little attention to indication and/or treatment duration [123,124]. Antimicrobial stewardship (AMS) programs are designed to improve the quality of prescribing practices in terms of choice of antibiotic, dosage, duration, route of administration and de-escalation. Some studies have shown the impact of AMS on the delay for adaptation of antibiotic therapies in patients treated for bloodstream infections [125] and on the duration of antibiotic therapy. However, the issue of whether AMS programs may have a positive ecological effect on the gut microbiota is not answered yet. In addition to AMS, drug options to protect the gut microbiota are being developed. One-promising way to protect the gut microbiota is to develop molecules to chelate or degrade unexpected residual antibiotics in the colon, thus limiting their impact on gut microbiota. For example, ribaxamase (an orally administered beta-lactamase) and DAV-132 (delivering delivers a non-specific adsorbent which irreversibly captures antibiotics) are currently under development [126,127] (Figure 1).

### 7.2. Gut Microbiota Modulation as a Therapeutic Option

#### 7.2.1. Fecal Microbiota Transplantation (FMT)

The emergence of antimicrobial resistance (AMR) is an important concern for public health. Treatment of MDR infections is a major clinical challenge. Therefore, new solutions to control the colonization by difficult-to-treat MDR pathogens included a better knowledge of (i) microbiota-mediated mechanisms of antimicrobial resistance and (ii) modulation of gut microbiota by fecal microbiota transplantation (FMT) or selective digestive decontamination. FMT is a process by which the microbiota from a donor is transferred in the colon of a patient by either endoscopically or by oral administration of capsule preparations. Today, FMT is recognized as a clinically highly effective treatment for recurrent *Clostridioides difficile* infection [128], but FMT is also explored for other indications. Recently, FMT has been considered for the eradication of drug-resistant bacteria from their intestinal reservoir. Indeed, studies show that patients undergoing prolonged antibiotic therapy have a greater rate of antibiotic resistance genes in the microbiome compared to healthy adults [129,130]. In these patients, number and diversity of antibiotic resistance genes decreased after FMT [129,130]. However, it is important to also take into account that AMR genes can also be acquired from the FMT donor stool. Therefore, donor selection and transplantation standardization are urgently needed [131]. Many studies have evaluated FMT for MDR intestinal colonization. Nevertheless, only one randomized control trial has been published and showed a slight decrease of extended spectrum producing *Enterobacteriaceae* (ESBL) or carbapenem-resistant *Enterobacteriaceae* (CRE) carriage compared to controls when using non-absorbable antibiotics followed by FMT [132]. Finally, great variability in the studies in terms of (i) FMT indication (CRE, ESBL…), (ii) mode of delivery, (iii) donor selection (family, donor bank), (iv) type of selective digestive decontamination and (v) sample preparation demonstrate the lack of knowledge on the issue.

#### 7.2.2. Pre- and Probiotics

Prebiotics are non-digestible food components that have favorable effects selectively promoting the proliferation and/or activity of one or more species of bacteria in the colon. Probiotics, on the other hand, are isolated viable organisms administrated to confer a health benefit on the host.

These products could reconstitute altered gut microbiota by promoting recolonization by some species either through the indirect effect of prebiotics or through a judicious choice of bacterial species for probiotics.

##### Prebiotic

Human milk oligosaccharides (HMO), which are an important component of breast milk, are one example of a prebiotic. HMO is known to help restore the balances between *Firmicutes* and *Bacteroidetes* in healthy subjects following antibiotic therapy exposure [133]. Furthermore, a randomized study on the clinical efficacy of a synthetic oligosaccharide in the decolonization of patients colonized with MDR bacteria is recruiting (VITORA study, NCT03944369).

Recent literature suggests dietary factors can alter the gut microbiota and may play a role in the risk of infection by gut pathogens [134]. Dietary fiber appears promising in promoting a diverse, healthy gut microbiota by selecting for fiber-degrading microbes that produce immune-enhancing compounds like butyrate [135]. The Winning the War on Antibiotic Resistance (WARRIOR) project is a study examining associations of dietary fiber consumption with the composition of the gut microbiota and gut colonization by MDROs [136].

##### Probiotic

The first studies evaluating the impact of probiotics included patients with cancer and focused on minimizing adverse effects following chemotherapy and radiation, such as severe diarrhea. In 2018, a Cochrane review in these patients found only 25% of studies demonstrating the efficacy of probiotics in preventing severe diarrhea [137]. Furthermore, some studies have even reported probiotics-associated morbidity and mortality [138,139]. Probiotics with bacteria that excel as gut colonizers are highly attractive agents. Two randomized studies reported success in the decolonization of patients with vancomycin-resistant enterococci using *Lactobacillus rhamnosus GG* [140,141], whereas the combination of *Lactobacillus bulgaris* and *Lactobacillus rhamnosus* had no effect on the colonization rate in the Gram-negative range [142].

## 8. Conclusions

The human gut microbiota plays an important role in the acquisition and conservation of a colonization by MDR pathogens. Therefore, it seems important to take into account the ecological impact of the antibiotics we prescribe, to develop strategies to limit the negative impact of antibiotics on the gut microbiota and to explore ways to restore its diversity in case of dysbiosis. While AMS is already receiving growing worldwide recognition as an interesting approach, other strategies (pre and probiotics, selective digestive decontamination, FMT) are still under preclinical or clinical evaluations.
